# Supplementary material for: Demographics shape public preferences for carbon dioxide removal and solar geoengineering interventions across 30 countries
Source: Commun Earth Environ. 2024 Oct 29;5(1):642. doi: 10.1038/s43247-024-01800-1 (PMC11522002; doi:10.1038/s43247-024-01800-1)
Supplement: Supplementary file 2 — Supplementary Information [file 43247_2024_1800_MOESM2_ESM.pdf]

Supplementary Tables

**Supplementary Table 1**: List of countries and language in which survey was conducted

| Country | Language |
|---|---|
| United States | English, Spanish |
| United Kingdom | English |
| Australia | English |
| Canada | English, French |
| Austria | German |
| Germany | German |
| France | French |
| Switzerland | German, French, Italian |
| Sweden | Swedish |
| Denmark | Danish |
| Norway | Norwegian |
| Estonia | Estonian |
| Poland | Polish |
| Greece | Greek |
| Italy | Italian |
| Netherlands | Dutch |
| Spain | Spanish |
| Chile | Spanish |
| Dominican Republic | Spanish |
| Brazil | Portuguese |
| Japan | Japanese |
| China | Chinese Simplified |
| Singapore | Chinese Simplified, English |
| Indonesia | Indonesian |
| India | Hindi, English |
| Turkey | Turkish |
| South Africa | English |
| Kenya | English |
| Nigeria | English |
| Saudi Arabia | Arabic, English |

**Supplementary Table 2**: Overview of Survey Design

| Procedure | Related measures |
|---|---|
| Screening questions | Age, Gender, Geographic area, Region |
| *Introductory text on CDR and SRM* | |
| *Randomized assignment to technology grouping*:<br>(1) Solar Radiation Management; (2) Carbon Dioxide Removal 1 (nature-based); (3) Carbon Dioxide Removal 2 (engineered) | |
| Comprehension questions – 2 items | |
| Familiarity with technologies | *Familiarity* – 1 item per technology |
| Assessment of perceived risks/benefits | *Perceived risks* – 4 items per technology<br>*Perceived benefits* – 4 items per technology (adapted from Jobin and Siegrist (2020), based on Wright et al. (2014)) |
| Ranking of risks | *Risk ranking* – ranking of up to 4 risks (for SRM) and 5 risks (for CDR1, CDR2) |
| Weighing risks and benefits | *Risk-benefit weight* – 1 item per technology (adapted from Pidgeon and Spence 2017) |
| Assessments of support for technology | *Perceived support* – 3 items per technology (1 on research and development, 1 on small-scale field trials, 1 on broad deployment), adapted from Pidgeon and Spence (2017) and Jobin and Siegrist (2020) |
| Assessment of policy support | *Policy support* – choice of up to seven policies at international or domestic level |
| Assessment of potential covariates | *Aversion to tampering with nature* – 5-item scale, developed by Wolske et al. (2019)<br>*Environmental identity* – 3-item measure adapted from van de Werff et al. (2013)<br>*Trust in institutions and science* – 5-item measure, adapted from Jobin and Siegrist (2020)<br>*Sources of information* – choice of up to nine information sources as credible<br>*Affect related to climate change* – 4 items, adapted from Feldman and Hart (2021)<br>*Concern over climate change* – 3 items, adapted from Steentjes et al. (2017)<br>*Beliefs about climate change* – 2 items<br>*Ownership of green products* – 2 items (1 on solar panels, 1 on electric vehicles |
| Demographics | Occupation, Education, Income, Religiosity, Political views, Member of minority or indigenous group |
| End Question and Debrief | |

**Supplementary Figure 1**: Background Information

The negative effects of climate change are becoming more apparent.

Caused by the human-driven release of carbon dioxide (CO2) and other greenhouse gases, the Earth's temperature is increasing, sea levels are rising, and extreme weather events are happening more often.

Measures to help limit the effects of climate change have been proposed, including reducing how much greenhouse gas is emitted (mitigation) and preparing for the current and predicted impacts of climate change (adaptation).

The current survey focuses on two more suggestions: carbon dioxide removal and solar radiation management. The first aims to remove CO2 from the atmosphere, before storing in plants, underground, or at the bottom of the ocean. The second aims to reflect how much sunlight reaches the Earth in order to reduce global temperature levels.

In the rest of the survey, we would like to know what you think about a few of these measures.

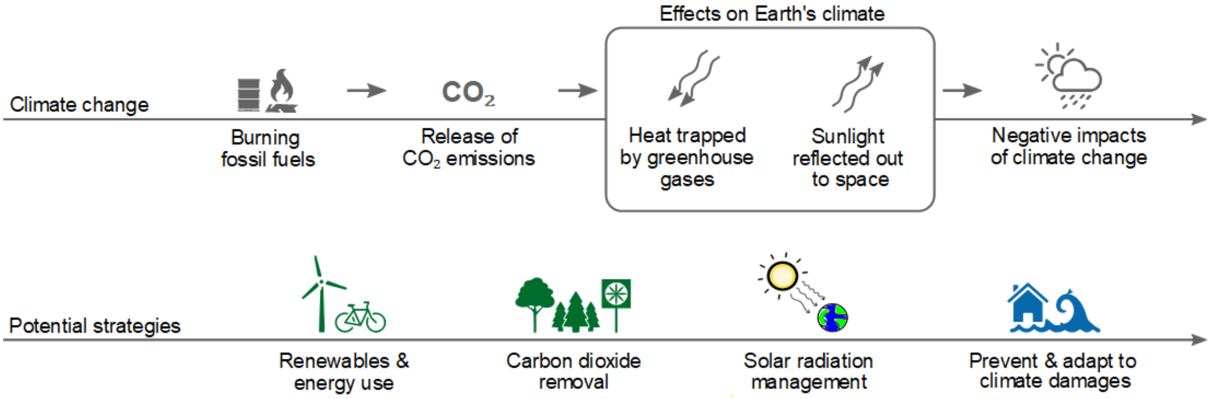


**Supplementary Figure 2**: Information Texts for all Climate-Intervention Technologies

We will now provide you with some background information on a few technologies. In the remainder of the survey, we want to get your feedback on these technologies, so please read the texts carefully.

To make sure you have understood them, the next slide will have one or two short questions to see how well you have understood. In order that you do not feel the need to rush, you will only be able to click to the next slide after 15 seconds have passed.

*Group 1 (SRM)*

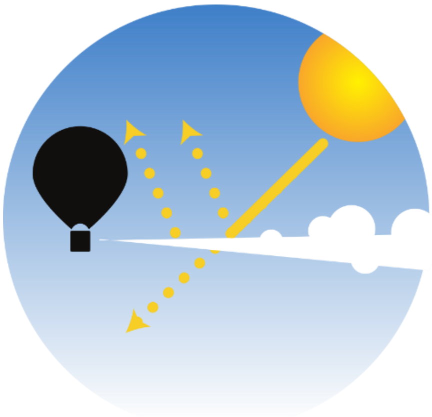

*Stratospheric Aerosol Injection* – This aims to limit the effects of climate change by using planes or balloons to spray small particles (aerosols) into the upper atmosphere.

The particles would reflect sunlight back into space. This could cool temperatures on Earth. But for this idea to work, we would have to keep doing it continuously. If we stopped, temperatures would rise once again, and probably very quickly. This would not do anything to reduce our greenhouse gas emissions or help with other impacts, such as ocean acidification.

*Marine Cloud Brightening* –

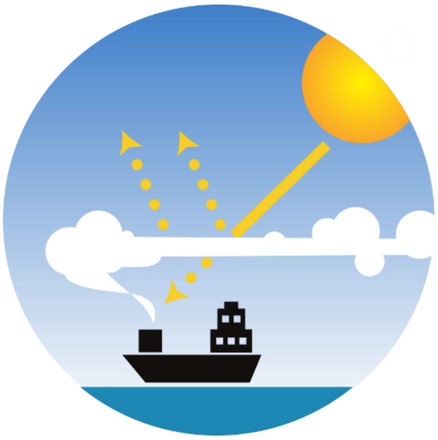

This aims to limit the effects of climate change by spraying small particles, such as sea salt, into the air over the oceans, to make clouds brighter.

These clouds would reflect sunlight away from the Earth, which could cool temperatures on a local or regional level. Marine cloud brightening might also help to protect ecosystems threatened by climate change. One way to spray the particles would be to use a fleet of ships. But for it to work, we would have to keep doing it continuously. If we stopped, temperatures would rise once again. Also, marine cloud brightening would not do anything to reduce greenhouse gas emissions or help with other impacts, such as ocean acidification.

*Space-based Geoengineering* –

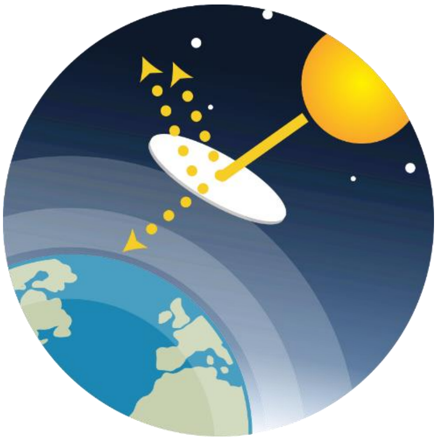

This aims to limit the effects of climate change by putting a giant mirror or other reflective material in outer space between the Earth and the sun.

Such a space mirror or sunshield would deflect sunlight back into space. This could cool temperatures on Earth. This would avoid direct changes to the land, oceans, or atmosphere of the Earth itself. But for this idea to work, we would need to be able to build and maintain something in space much larger than ever before. A space mirror would also be very costly to build, given that its location would be about four times as far from the Earth as the Moon. Also, it would not do anything to reduce our greenhouse gas emissions or help with other impacts, such as ocean acidification.

*Group 2 (CDR1)*

***Afforestation and Reforestation*** –

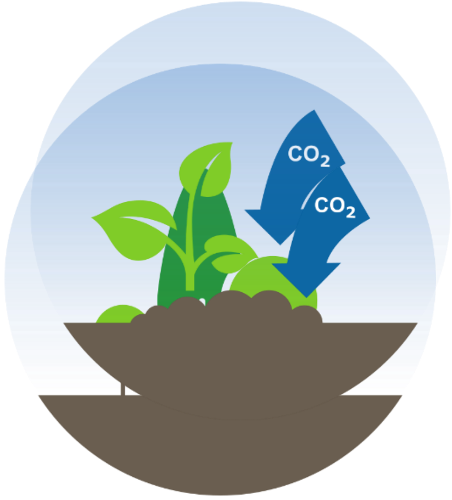


Both aim to limit the effects of climate change by planting trees.

As trees grow, they absorb carbon dioxide from the atmosphere and store it for decades or longer, as long as the forest is around. But for this idea to work, we would need a lot of land and water. It is therefore likely to compete with agriculture and other uses. Also, if trees were cut down or happen to burn down, then the carbon dioxide would again be released into the atmosphere.

***Soil Carbon Sequestration*** –

This aims to limit the effects of climate change by changing agricultural techniques to store more carbon dioxide in soils.

This could include planting different crops, leaving crop residues on the field, or increasing the number of trees on agricultural lands. By improving soils, it is possible to remove carbon dioxide from the air and store it in soils for decades to centuries. Soil carbon sequestration also requires no additional land and might make soils better for farming. But for it to work, we would need farmers and people in other industries to cooperate and take part. Also, if agricultural practices are not sustained, the carbon dioxide would again be released into the atmosphere.

***Marine Biomass and Blue Carbon*** –

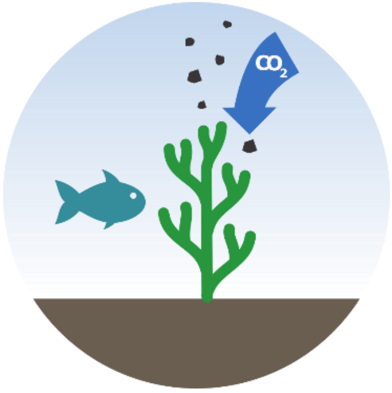


Both aim to limit the effects of climate change by improving how much carbon dioxide is stored in the oceans.

Blue carbon does this by restoring or growing ecosystems such as mangroves, salt marshes, and seagrass meadows. Marine biomass does this by growing seaweeds or macroalgae. All of these absorb carbon dioxide from the atmosphere as they grow. This can then be stored for decades to centuries at the bottom of the ocean. But for this idea to work, we would need many people, especially those in coastal communities, to cooperate and take part.  Also, if ecosystems are disturbed or destroyed or the plants are cut down, the carbon dioxide would again be released into the atmosphere.

*Group 3 (CDR2)*

***Direct Air Capture with Carbon Storage*** *–*

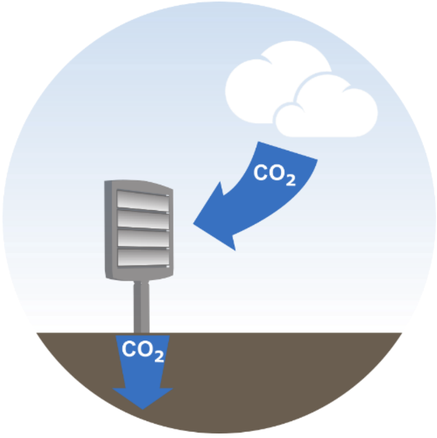


This aims to limit the effects of climate change by using very large fans to remove carbon dioxide from the air (direct air capture).

Once pulled into the fans, absorptive liquids convert the carbon dioxide using a chemical process. It can then be stored indefinitely underground (carbon storage). Direct air capture with carbon storage also needs little land. But for it to work, it would require lots of energy along with underground places to store carbon. Direct air capture is also extremely expensive right now and it is not clear if it works at the large scales needed, both of which limit how much it can be used.

***Bioenergy with Carbon Capture and Storage*** *–*

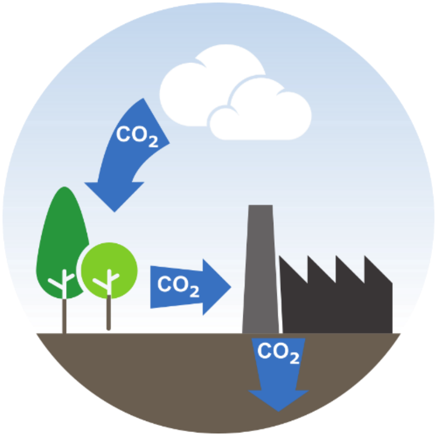


This aims to limit the effects of climate change by growing and harvesting plants as a source of energy (bioenergy).

As plants grow, they absorb carbon dioxide from the air. By burning these plants and chemically capturing the carbon dioxide released, bioenergy can provide energy for homes and businesses or be stored underground indefinitely (carbon capture and storage). But for it to work, we would need a lot of land and water (and underground places to store carbon). It is therefore likely to compete with agriculture and other uses. It is also not clear if bioenergy with carbon capture and storage will work at the large scales needed, though some industrial applications do already exist.

***Enhanced Weathering*** *–*

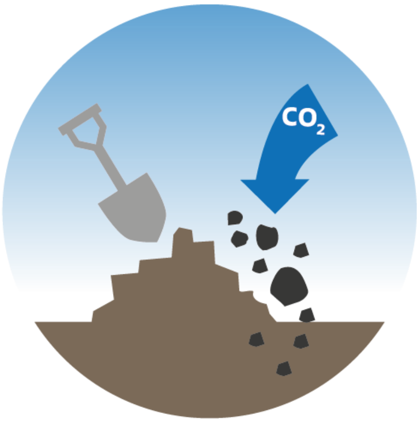


This aims to limit the effects of climate change by increasing the ability of rocks to absorb carbon dioxide from the atmosphere.

As rocks such as limestone and basalt are exposed in nature to processes like rain, wind, or waves, they are ground down (weathering), which allows them to absorb carbon dioxide. Since this process takes place extremely slowly, enhanced weathering speeds it up by physically or chemically grinding the rocks before placing them onto soils, beaches, or next to rivers. Over time, rocks and their carbon dioxide are ultimately stored in oceans indefinitely. But for it to work, we would need a lot of rocks. This could cause negative ecological and human health impacts (and greater energy use) from more mining and extraction. Also, it is not clear if it will work at the large scales needed, as only limited trials have been done so far.

***Biochar –***

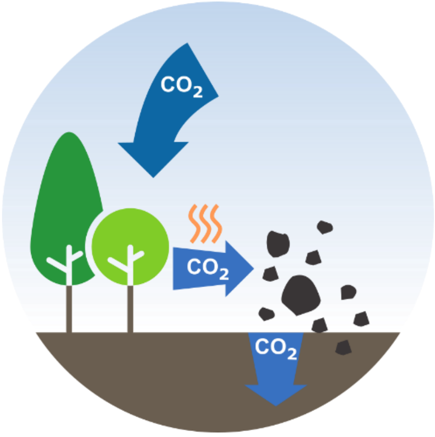


This aims to limit the effects of climate change by heating organic material, such as tree branches and cornstalks, inside a container with no oxygen.

This creates black material very similar to charcoal (biochar). If we grind this up and add it to soil, it is possible to remove carbon dioxide from the air and store it in soils for decades to centuries. Biochar might also make better soils for farming. It could also be added to other things, such as concrete, animal feed, or compost. But for it to work, we would need farmers and people in other industries to take part and, potentially, change how they do things. We would also need a lot of organic material. Biochar is also quite expensive right now, which limits how much it can be used.

Methods and Survey Description

## Languages and Translation

The survey and all related information provided to participants were translated (by professional translators) into the language(s) most predominantly spoken in target countries. In total, this entailed the survey being translated into a total of 19 languages. For countries which are deeply multilingual and where many languages were spoken by the different constituent ethnic groups (e.g., India, Kenya, Nigeria), we employed the dominant *lingua franca*, typically English. After soft launch of the survey in these countries, examination of the sample composition as well as a check of individual comments in available open-ended questions satisfactorily ruled out use of English presenting any difficulties. In India, however, soft launch revealed that reliance on English resulted in apparent restrictions on which individuals could take part. Data collection was thus paused to allow for all materials to be translated to Hindi, with the survey then opened back up and individuals allowed to choose whether they wanted to take part in English or Hindi. A similar choice was given to participants in five other countries where (at least) two languages were spoken by large subsets of the population, to encourage their participation in the language that best suited them – for instance, those in the United States could choose between Spanish and English, those in Canada between English and French, and those in Switzerland between Italian, French, and German (Appendix A.1). All of this was intended to facilitate as broad and deep participation in the survey (and topic) as possible. Furthermore, in view of sensitivities around certain questions in some countries, all questions about educational attainment, income levels, and so on were subjected to processes of "localization" before being launched. This was done with strong appreciation of how words, concepts, and even emotions are expressed in the Spanish of Chile or Dominican Republic is different from that in the Spanish of Spain. In this manner – also in the case of the countries in which the survey was presented in German, French, Italian, and Chinese Simplified – this ensured surveys would closely reflect the language used in the local context, and thereby avoid any confusion or uncertainty being introduced.

On the translation process, all materials were translated and proofread by separate professional translators who are native in the respective language and for all countries, before undergoing an additional review by another linguist along with "quality assurance" by internal teams at the translation agencies. At Norstat, there was an additional check, where available, by an internal native speaker who checks the accuracy of translation once more. This is then followed by two more project managers (or executives) reviewing to ensure all the overlays have been correctly applied and generally ensuring overall accuracy. As a final step, one of the co-authors carried out trial runs with both survey versions (mobile and desktop) for all languages, with this being done by a co-author native in the language if possible or, at a minimum, employed translation tools. In total, survey translations were screened and pre-tested by four or five, (and often more) individuals, from the research team and at Norstat. Such checks were conducted (i) for desktop and mobile versions to ensure there were no issues with formatting of overlays, i.e., to avoid issues, especially for "longer" languages such as German or Japanese, of text not fitting into boxes. These checks (ii) also involved inspection of the translations, e.g., to ensure there were no inconsistencies with how specific technologies or concepts were described in the survey and to confirm that

professional translators employed the suggested keyword translations from the native-language experts. Given the specialist nature of the topic – and possible concerns certain words may be difficult for those without expertise in the field to translate – a list of 21 keywords (e.g., geoengineering, carbon sequestration, climate mitigation, direct air capture) was prepared for a network of native-language experts who worked in the field to translate. The list, along with any additional comments, were forwarded to translators to facilitate and guide translation activities. In general, this underscores the difficulty of translating potentially novel terms that are not widely known or wholly unknown in the target language – and which may thus have to assume a "transliterated" form, i.e., by translating the words without trying to fit them into the context of the language itself – along with the importance of engaging with local experts, where available. In cases where there was a divergence between the translations of the topic experts and the professional translators, e.g., for French, Danish, and Norwegian, this was subjected to back-and-forth discussion over why a decision was taken and thereby resolved. As an example, translation of "deployment" in Danish and Norwegian was discussed, and ultimately changed, to better reflect the initial understanding in English rather than the sense of "application" (in Danish: "Udrulning" versus "Udbredelsen"). There were similar situations in French leading to, e.g., changing one of the items for the *aversion to tampering with nature* scale so that it was described as a "downfall" (*chute*) rather than a "loss" (*perte*). In the case of Chile (Spanish), the translation of one of the items on risks and benefits was initially translated to convey it "… can be safe if done in a controlled way" rather than "can be done safely in a controlled fashion." Accordingly, this was changed in Spanish to: "… se puede hacer de forma segura y controlada". Furthermore, there were extensive revisions to the German translations, which were resolved through back-and-forth discussions the translators to ensure texts were comprehensible, easy to read, and moreover aligned with the English originals. By way of an example, for "marine cloud brightening", a description was changed from "…in order to make clouds *whiter*" to "… in order to make clouds *brighter*" (i.e., "…um die Wolken *weißer zu machen*" to "... um die Wolken *aufzuhellen*"), Similarly, the translation for "enhanced weathering" was changed from "*verbesserte* Verwitterung" (i.e., *improved* weathering) to "*beschleunigte* Verwittering" (i.e., *accelerated* weathering). In this vein, where disagreement specifically involved translations of technologies, we searched to see whether one version already appeared in the target language, in which case this one would be used. In general, we also tended to include the English term in parentheses next to the translation, with this also suggested by many of the topic experts. Overall, the steps undertaken underscore the efforts made to achieve the best possible translation.

**Information Provision**

After initial screening questions, we provided all participants with background information via a short text (and graphic) on carbon dioxide removal and solar radiation management (Supplementary Figure 1). This text situated these measures in relation to the negative effects of climate change and, to avoid providing a sense that these solutions were the only ones possible or in any way a kind of panacea, also explicitly referred to mitigation and adaptation as other options. By including all of these in the graphic as well, we aimed to further avoid such a priming being imparted. In order to make sure participants took time to engage with the text, they were required to spend at least 20 second on the page before clicking further.

After the background information, individuals were informed that they would now be provided with information on a few technologies. To avoid careless reporting, which can particularly be an issue in online surveys (Ward and Meade 2023), we asked that they "please read the texts carefully" as "we want to get your feedback on these technologies". In addition, they were told that their understanding would be checked by one or two short questions and that going faster would not be possible: "In order that you do not feel the need to rush, you will only be able to click to the next slide after 15 seconds have passed."

At this point, individuals were randomly assigned to one of three groups, corresponding to the technology categories: SRM; nature-based CDR; engineered CDR. We opted for this approach so that participants would have to jointly evaluate multiple technologies. At the same time, in order to reduce cognitive load on participants and allow them to engage with the informational materials as much as possible, the groups were composed so that they would be broadly similar to one another, or at least to avoid significant differences. Differences were specifically avoided so that, *inter alia*, perceptions of risks and benefits and overall assessments of the technologies would not be inflated in one direction or another due to their being seen as overly different. For instance, if afforestation and reforestation, DACCS, and space-based geoengineering were grouped together, it was adjudged that having to read and understand information on novel and quite different technologies could (a) be more challenging for participants, thereby affecting engagement and (b) cause individuals to use the most familiar of the technologies, afforestation and reforestation, as an "anchor" when evaluating the others (Tversky and Kahneman 1974). Participant responses for the others would thereby be overly subject to the particular context in which this decision was taken. Indeed, other authors (e.g., Jobin and Siegrist 2020) have opted to randomly present only one technology to each person in order to avoid such compositional bias. However, doing so would lead to substantial reductions in the sample size for each of the options – indeed, the effective sample size of Jobin and Siegrist (2020) is around 150 for each technology – and would rule out exploring how individuals evaluate multiple technologies. We view such insights as particularly important given the growing discussion of the need to employ a portfolio of climate-intervention technologies to limit effects of climate change (MacMartin et al. 2018; Sovacool 2021; Ricke and Moreno-Cruz 2022) as well as the growing emergence of projects and field trials that employ a combination of options to leverage potential synergies (see Low et al. 2022). Similar to Cox et al. (2020) and Sweet et al. (2021), we therefore opt to present multiple technologies to individuals for their consideration, arguing that using clusters of technologies pre-identified from the literature (Low et al. 2022, Dooley et al. 2021) allows us to avoid the shortcomings mentioned above. As a result, the three technologies (stratospheric aerosol injection, marine cloud brightening, space-based geoengineering) formed one cluster, and the seven CDR technologies were grouped by more nature-based approaches (afforestation and reforestation, soil carbon sequestration, marine biomass and blue carbon) and rather more engineered approaches (enhanced weathering, biochar, DACCS, BECCS).

Each information text for the climate-intervention technologies followed the same format (see Supplementary Figure 2), by starting with a broad description of how they would (or do) work, followed by 2-3 sentences of more detail and potential benefits, and then 2-3 sentences around prospective risks, and through use of pictures employing the same graphic style. All pictures were designed by the same graphic designer and so that they would all have a similar

quality and tone. Designs for some of the pictures were iteratively revised, whether to correct any inaccuracies in what was conveyed or so that participants could understand the content more easily. Regarding the information texts, these always began with a sentence about how the measure aimed to "limit the effects of climate change", followed by more detail of how the method worked, while using language as straightforward as possible. Finally, each of the texts concluded with a sentence or two, usually beginning with "However" that mentioned some of the downsides or uncertainties with the technology. Here, we oriented discussions around the negative impacts listed in Fuss et al. (2018), Part 2, Table 2, thus with one main socio-economic and one main environmental risk – the same approach was also tailored to the SRM options. In this way, we could be more systematic in our presentation of potential risks while striving to gather insights on the type of risks which were of greatest concern to participants. At the same time, rather than enumerating the benefits and risks of the technologies, we instead attempted to provide a description of the techniques. As such, discussions of potential risks and benefits were intended to be illustrative rather than exhaustive, and with the overall aim here to offer participants enough information to feel as if they could reasonably assess the technologies. One consideration here is the finding that giving information about risks and benefits of climate-intervention technologies, rather than providing a description of the technology, tended to result in reduced support (Braun et al. 2018; Wolske et al. 2019). Such "hint of risk" effects are quite common for perceptions of unfamiliar technologies and media coverage in general, where a more detailed discussion of a subject can elicit a negativity bias across responses (Gregory and Lichtenstein 1994; Rozin and Royzman 2001; Satterfield et al. 2023). Information texts were devised to focus on highlighting how the technologies would (or do) work, rather than to provide a detailed account of risks and benefits.

One resulting limitation, however, is that despite best efforts to ensure the structure, substance, and format of the information texts was consistent for all technologies, unintended differences in complexity may have emerged during the pre-testing and piloting process. In specific, given inherent differences in the complexity of the technologies themselves and their familiarity for participants (contrast "Afforestation and reforestation" with "Space-based geoengineering"), there are differences in the length of some texts, often in response to participant feedback, even where such differences were avoided as much as possible.

In retrospect, however, it might be that this balance of remaining as "neutral" as possible may at times have missed the mark in providing accurate characterizations (in short text form) of the technologies. Probably the clearest example is "stratospheric aerosol injection", given that this text is among the shortest and does not place substantial emphasis on possible impacts on the biosphere and weather patterns. Here, we would note that we decided to omit mention of specific impacts such as the harmful effects on monsoon or droughts since such specificity was not present for any other technology, and given that there is still a degree of uncertainty around this effect (Da-Allada et al. 2020; Krishnamohan and Bala 2022; Liu et al. 2023). In addition, this text, probably more than the others, was subject to extensive revision to simplify its presentation, such that its resultant lack of "complexity" is itself the subject of the pre-testing process. Still, we acknowledge that, by trying to provide a balanced presentation of risks and benefits and to provide participants broad information from which they could draw their own inferences, in this case, it may have unintentionally resulted in an insufficiently comprehensive illustration of potential risks. Nonetheless, it is interesting that,

even with such limitations in mind, stratospheric aerosol injection was the least supported of the options, both in the Global North and Global South. As such, while we acknowledge the foregoing, it does not ultimately seem to be fundamental when it comes to the broader insights of this research.

**Quality Checks and Participant Replacement**

Multiple steps were taken to ensure the final dataset was of high quality, specifically through several quality checks and, ultimately, by removing and replacing respondents which failed to satisfy a range of criteria. First, there were two comprehension checks though, based on results from the soft launch and given unfamiliarity of the topic, we opted not to exclude participants that failed to answer the question correctly in two attempts. Conversely, if someone answered the second (true/false) question wrong twice, then were removed, taking this as a good indicator of lack of attention. Second, there were two trap questions included in the survey, e.g., where participants were instructed to select "Strongly agree" – if individuals answered both questions incorrectly, they were also removed. Ultimately, we opted against removing participants if they only answered one trap question wrong given that the survey entailed lots of novel information, which also could be quite complex. Third, a "speeder flag" was instituted whereby participants who completed the survey in less than one-third of the median length of survey for a particular country were removed – in addition, for countries where the median length of survey was quite low, i.e., less than seven minutes, such individuals were also often removed and replaced, given questions of how realistic such a completion time would be given the minimum time constraints imposed for reading the information texts. Fourth, an open-ended question was included at the end of the survey, both to inquire about any possible problems and thereby make improvements to the survey and, in the case of problematic answers, as a reason to exclude participants. Lastly, at an administrative level, those with duplicate IP addresses and/or geolocation data which was, for whatever reason, not valid for a specific country.

In addition, all countries underwent a soft launch with 5-10% of the full sample to identify any potential issues, including with the translations, programming, and localization utilized to tailor the survey to the country context. These checks also considered whether changes were needed, e.g., to levels for income and education. Also, especially in view of the importance of issues of gender identity, we paid close attention to how the question about one's gender was framed in the languages. A handful of initial translations (e.g., Swedish, Norwegian') did not ask about gender but rather something to the effect of "Are you a man or a woman?". Translations were thus changed to a more neutral form, i.e., "What is your gender?". Similarly, in languages (like, German) where certain actors (e.g., climate activists or neighbors) can have both a masculine and feminine form, we made sure to include both of these in the answer option. In addition, as part of the soft-launch process, the data for every single country was assessed to make sure that (a) there was sufficient heterogeneity, specifically for the questions relating to perceptions of technologies, to determine if participants may be "straightlining" responses; (b) an inordinate amount of participants were not getting the trap questions and comprehension checks incorrect – for instance, if around 45% of participants in the SL data answered one of the trap questions incorrectly – we paused data launch to confirm with Norstat that all programming was working appropriately, that there were no issues with translation that could lead to confusion (done by a native speaker in the language), and also specifically reviewed the open-ended comments of the respondents

who failed this question to see if they flagged any issues; with such possibilities dismissed, and since those with incorrect answers would be “cleaned” from the final sample, we proceeded with data collection; (c) participants were not availing themselves too often for certain questions of “Prefer not to say” or “Don’t Know” or “Neither agree nor disagree”, not because the responses were themselves invalid or undesirable but, rather, to help identify if an item may not be working as expected in a given context and/or as a signal to keep an eye on something for full launch (or whether to change from an “unbalanced” to “balanced” scale; and (d) to identify early indications of the sample being unrepresentative in terms of age, gender, income, geographic region, and education. In one or two extreme cases, like for income in China, this led to changes in the income levels – otherwise, a closer eye was kept on quotas and patterns in responses to given questions. One final element of the soft launch data check considered the open-ended responses to the question included at the end asking if there are any issues or problems, which doubled as a “quality check”. Almost exclusively, such responses were of a kind expressing there was “no problem” or to “thank you for the survey”. In a couple instances, however, these helped to identify a potential problem early on, e.g., highlighting the need to use Hindi as a language for India or, more unexpectedly, to remove the question about whether one belonged to a minority group in Estonia. Upon reflection, this question seemed to be problematic for some survey participants given the Russo-Ukraine War and the presence of a sizable Russian ethnic minority group in Estonia. This question was thus removed for reasons of politically sensitivity and not wanting to irritate survey participants, as had also for instance been done for political views in China, with this question not asked in this country.

Supplementary Data and Figures

We provide here figures with the data for all three-way ANOVAs for each of the ten climate interventions as dependent variables. We provide figures for three-way ANOVAs using youth, gender, and poverty status as the factor variables; we then provide another set of three-way ANOVAs using youth, gender, and Global South vs Global North as the factor variables. The only figures missing from these 20 ANOVAs are the ones already presented in the main text of the article.

**Supplementary Figure 3:** SAI: Support for interventions by age, gender, and poverty

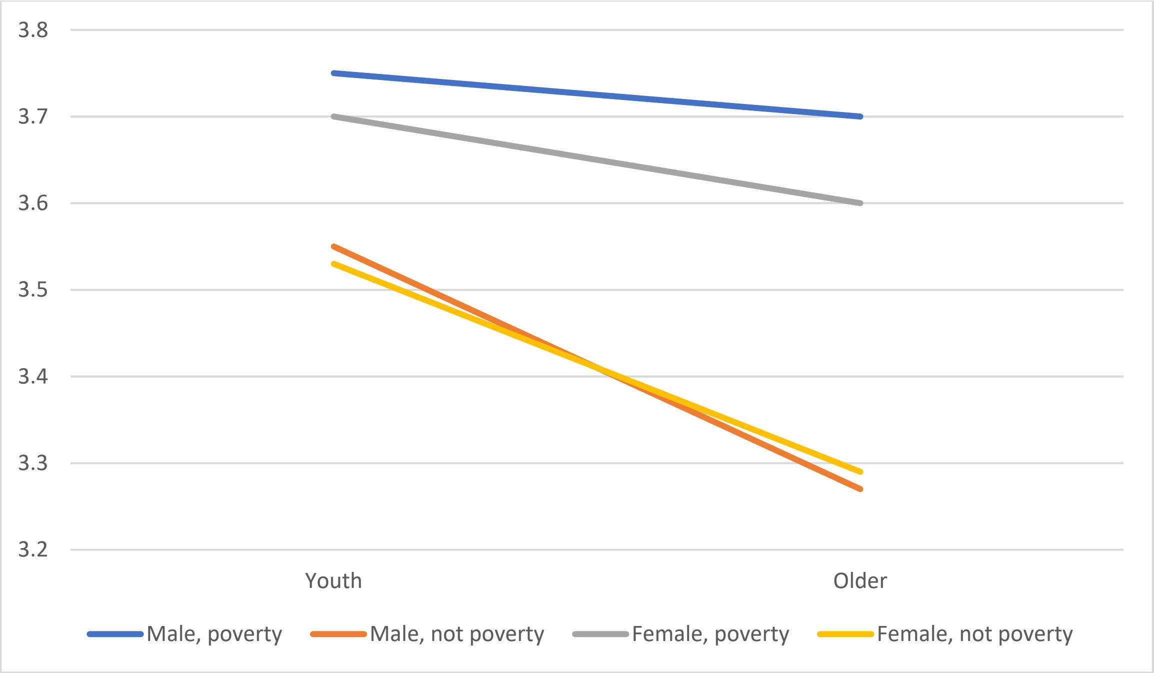


* In a three-way ANOVA, poverty and youth were significant, but gender, all three two-way interaction effects, and the three-way interaction were non-significant.

**Supplementary Figure 4**: Space: Support for interventions by age, gender, and poverty

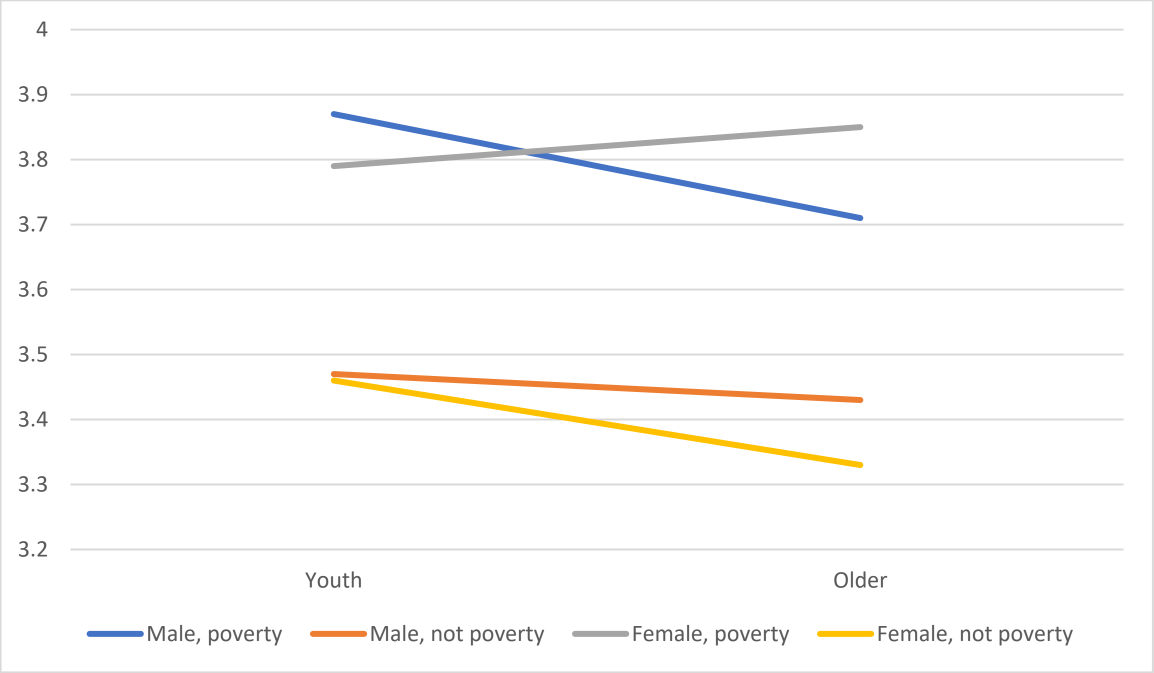


* In a three-way ANOVA, poverty was significant, but youth, gender, all three two-way interaction effects, and the three-way interaction were non-significant.

**Supplementary Figure 5:** Afforestation: Support for interventions by age, gender, and poverty

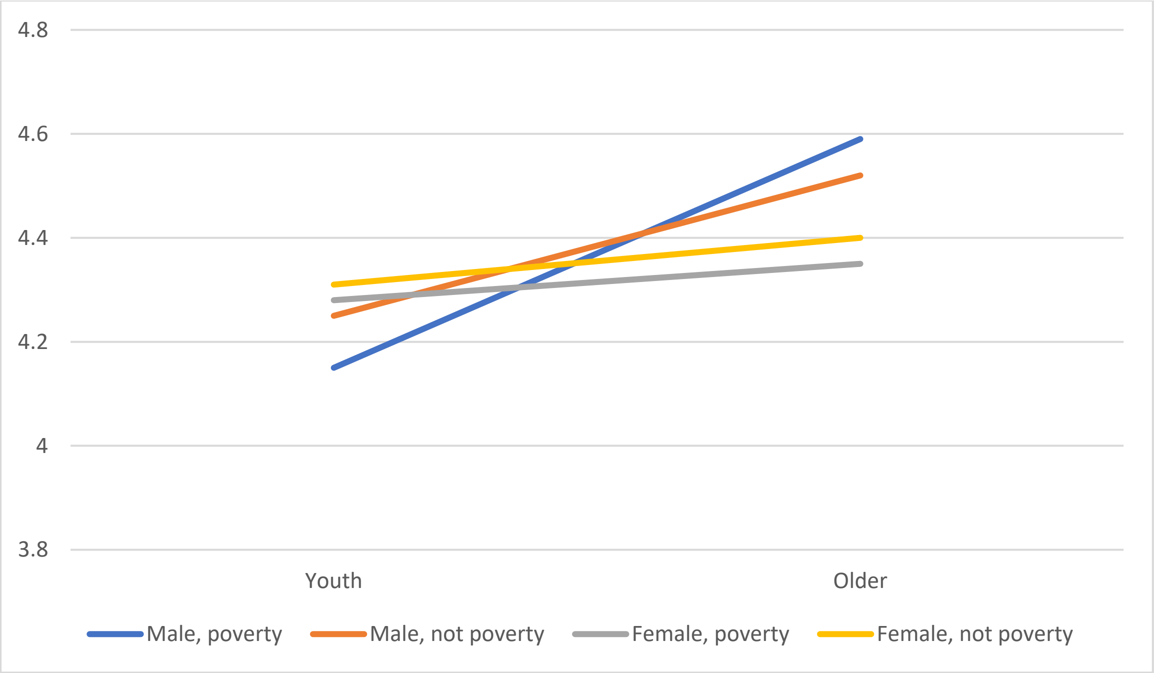


* In a three-way ANOVA, youth and the interaction between gender and youth were significant, but gender, poverty, the remaining two two-way interaction effects, and the three-way interaction were non-significant.

**Supplementary Figure 6**: Soil Carbon: Support for interventions by age, gender, and poverty

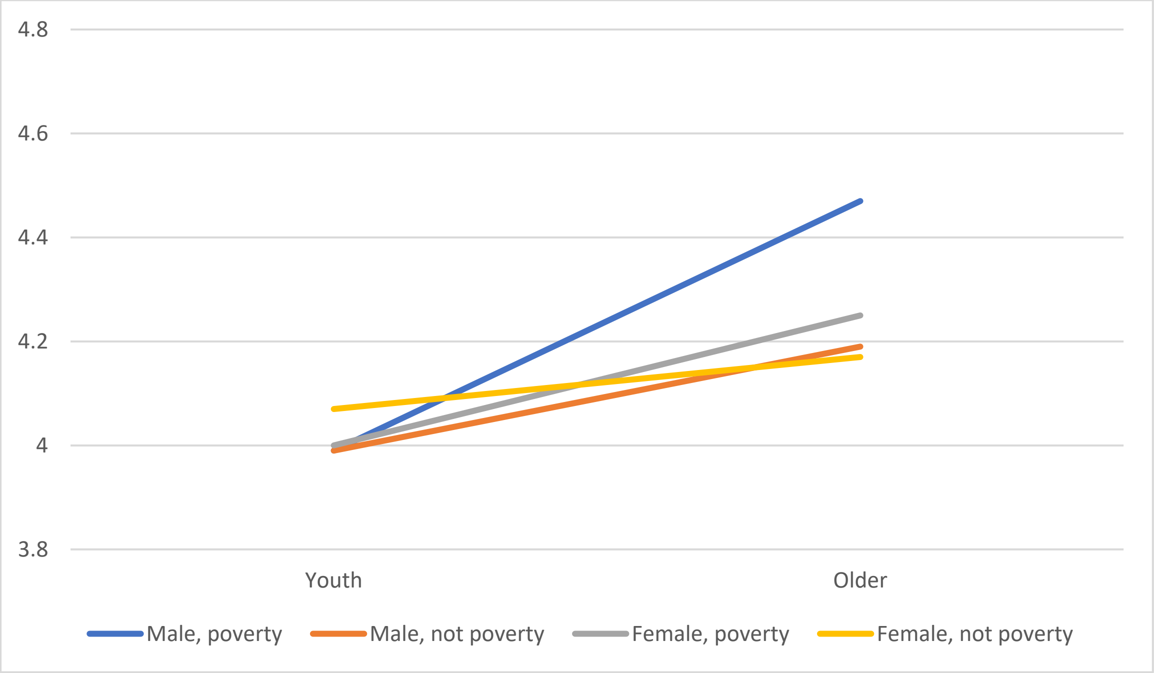


* In a three-way ANOVA, youth and the interaction between poverty and youth were significant, but gender, poverty, the remaining two two-way interaction effects, and the three-way interaction were non-significant.

**Supplementary Figure 7**: BECCS: Support for interventions by age, gender, and poverty

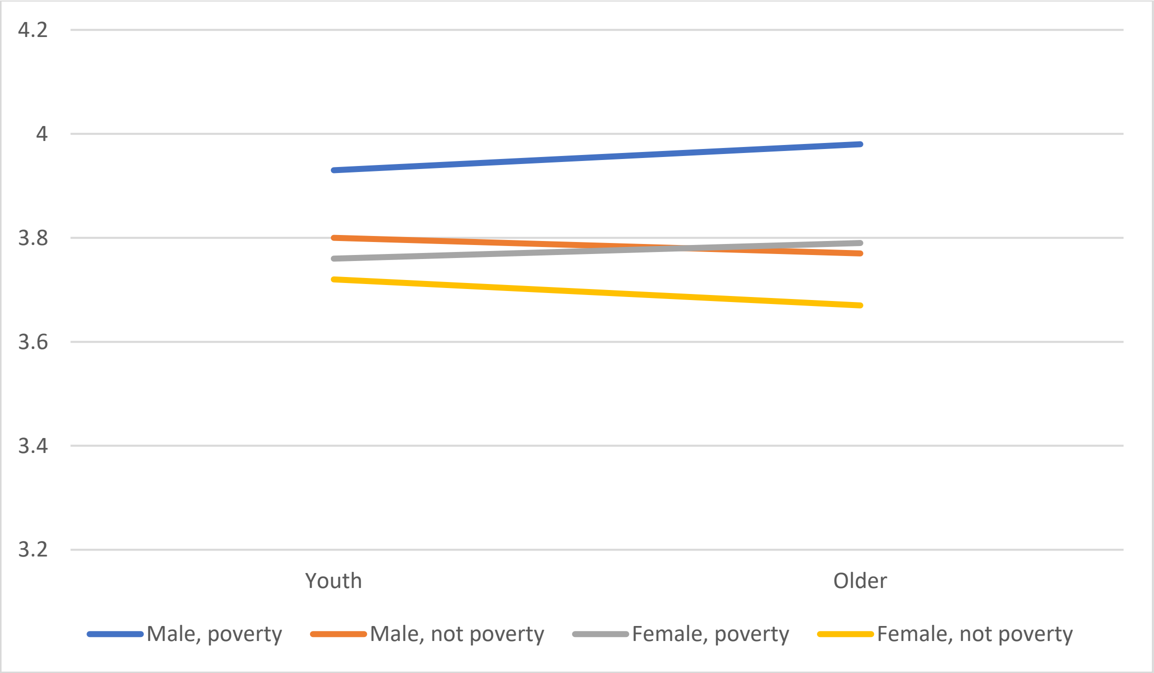


* In a three-way ANOVA, gender and poverty were significant, but youth, all three two-way interaction effects, and the three-way interaction were non-significant.

**Supplementary Figure 8**: ERW: Support for interventions by age, gender, and poverty

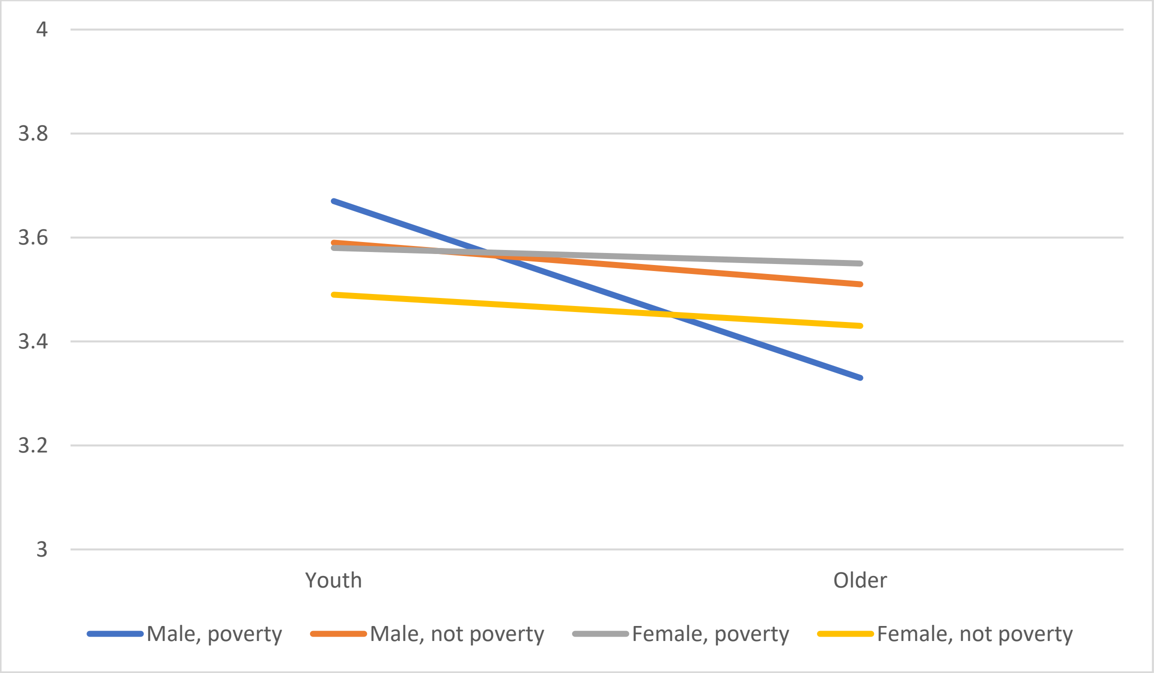


* In a three-way ANOVA, none of the three main effects or four interaction effects were significant.

**Supplementary Figure 9**: Biochar: Support for interventions by age, gender, and poverty

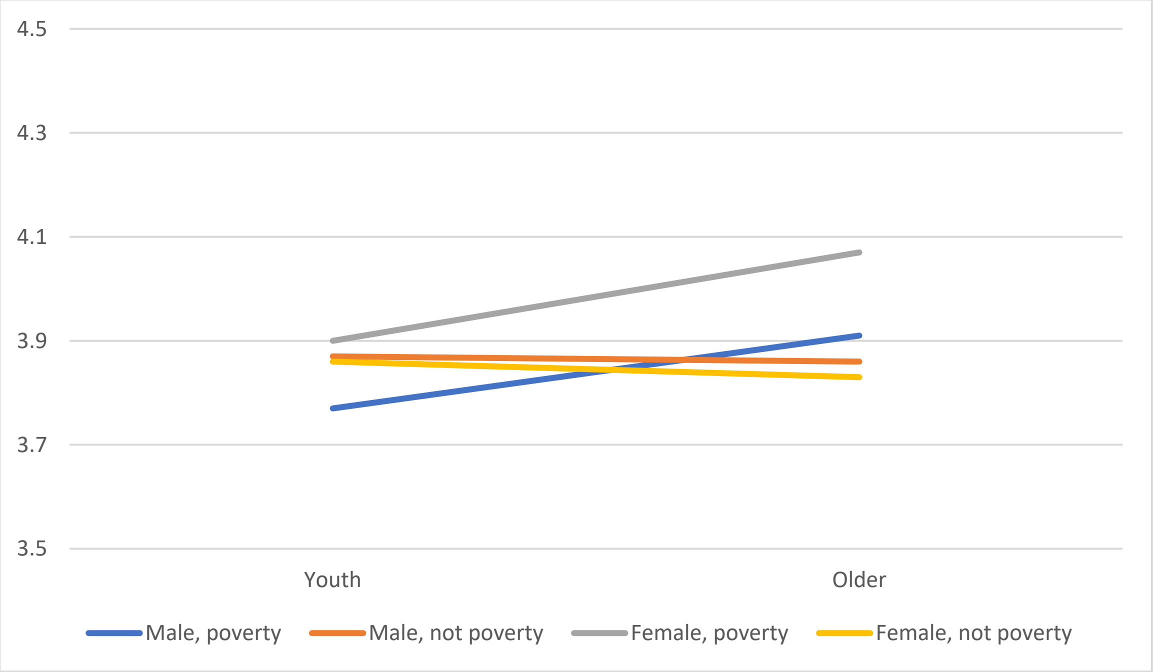


* In a three-way ANOVA, none of the three main effects or four interaction effects were significant.

**Supplementary Figure 10**: MCB: Support for interventions by age, gender, and Global South vs North

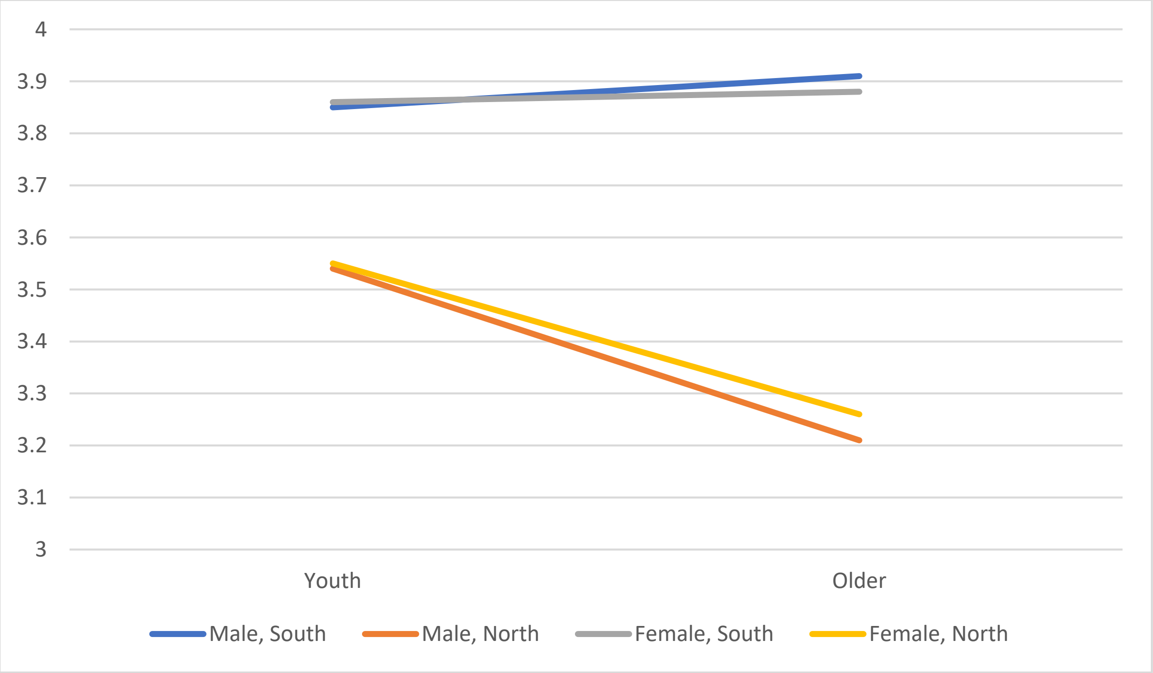


* In a three-way ANOVA, Global South, youth, and the interaction between Global South and youth were significant, but gender, the other two two-way interaction effects, and the three-way interaction were non-significant.

**Supplementary Figure 11**: Space: Support for interventions by age, gender, and Global South vs North

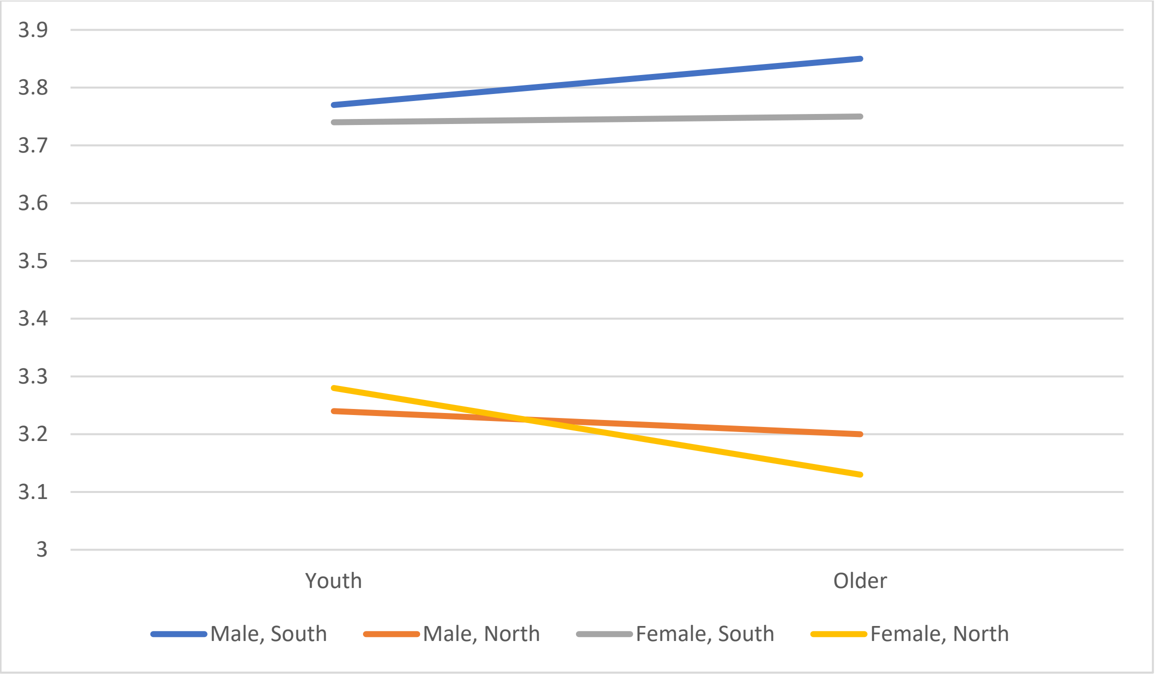


* In a three-way ANOVA, Global South and the interaction between Global South and youth were significant, but youth, gender, the other two two-way interaction effects, and the three-way interaction were non-significant.

**Supplementary Figure 12**: Afforestation: Support for interventions by age, gender, and Global South vs North

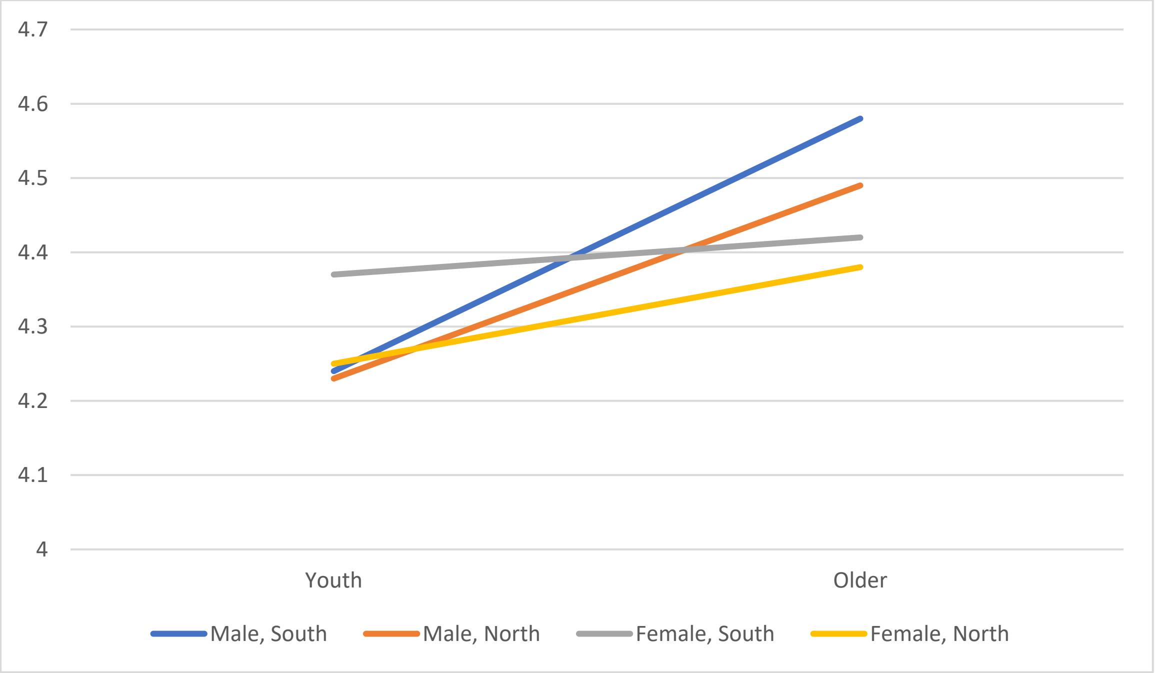


* In a three-way ANOVA, youth and the interaction between gender and youth were significant, but gender, poverty, the remaining two two-way interaction effects, and the three-way interaction were non-significant.

**Supplementary Figure 13**: Soil Carbon: Support for interventions by age, gender, and Global South vs North

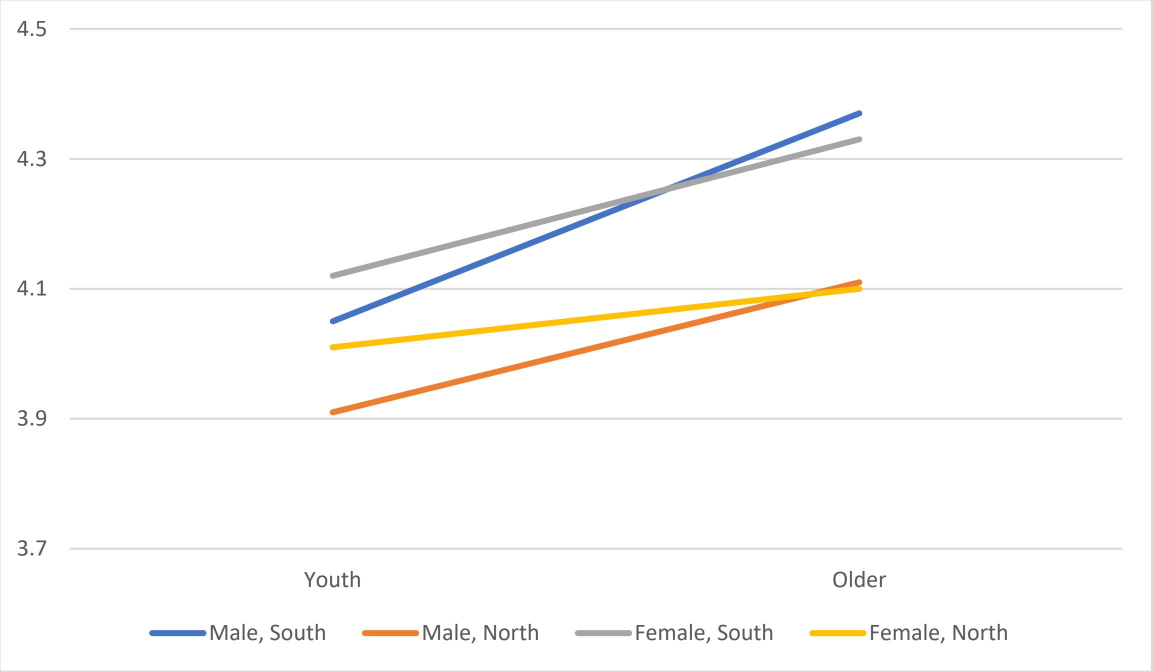


* In a three-way ANOVA, Global South, youth, the interaction between Global South by youth, and the interaction between gender and youth were significant, but gender, the remaining two-way interaction effect, and the three-way interaction were non-significant.

**Supplementary Figure 14**: Blue Carbon: Support for interventions by age, gender, and Global South vs North

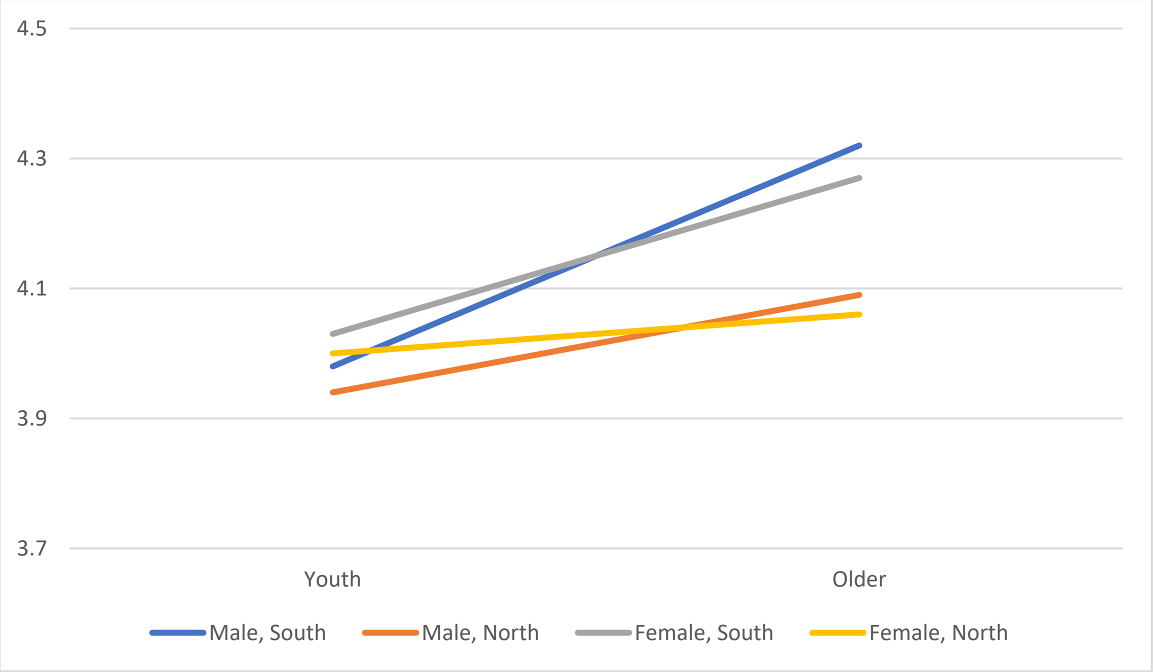


* In a three-way ANOVA, Global South, youth, the interaction between Global South by youth, and the interaction between gender and youth were significant, but gender, the remaining two-way interaction effect, and the three-way interaction were non-significant.

**Supplementary Figure 15**: DACCS: Support for interventions by age, gender, and Global South vs North

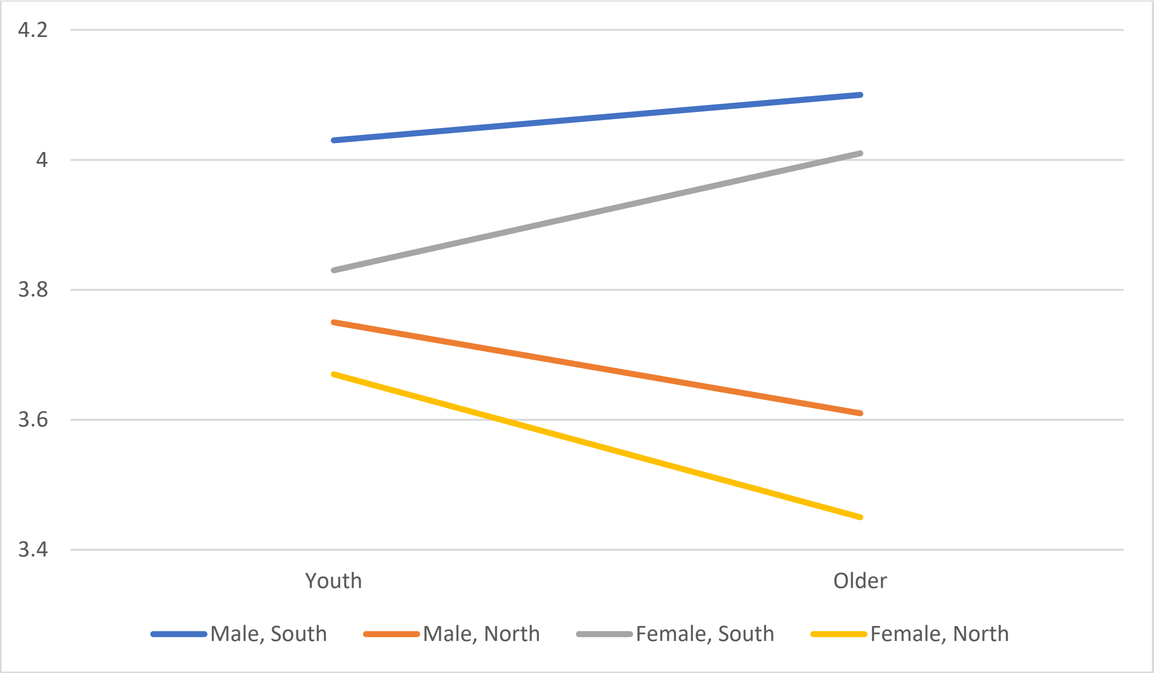


* In a three-way ANOVA, Global South, gender, and the interaction between Global South by youth were significant, but youth, the two remaining two-way interaction effects, and the three-way interaction were non-significant.

**Supplementary Figure 16**: BECCS: Support for interventions by age, gender, and Global South vs North

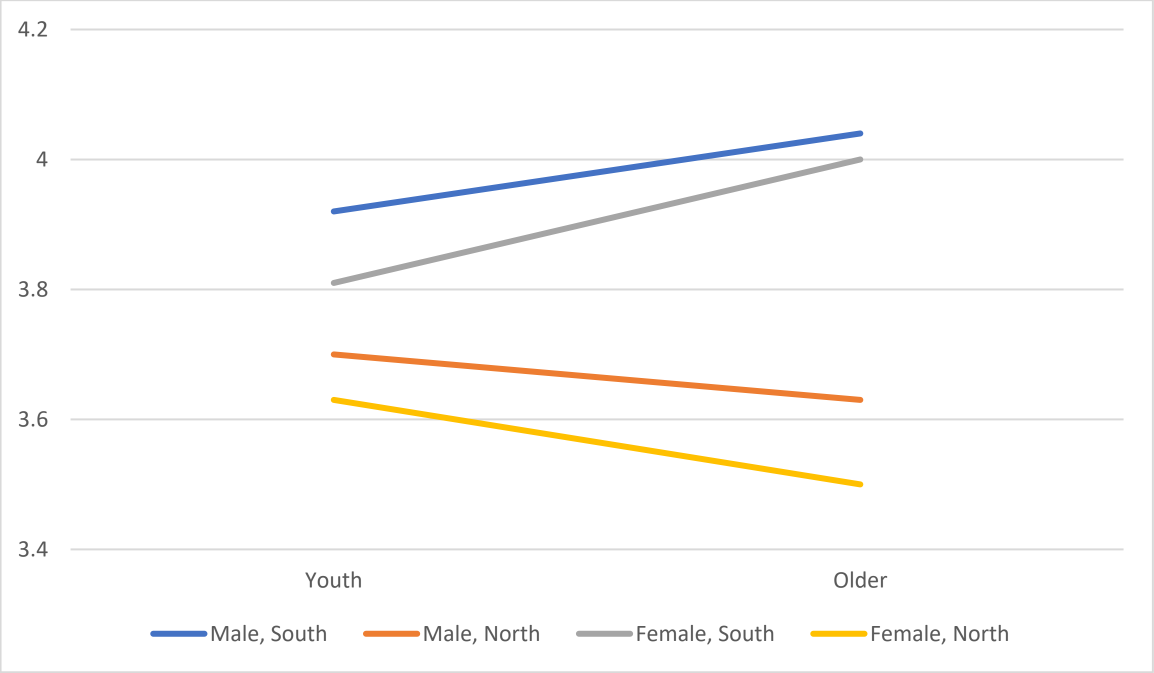


* In a three-way ANOVA, Global South, gender, and the interaction between Global South by youth were significant, but youth, the two remaining two-way interaction effects, and the three-way interaction were non-significant.

**Supplementary Figure 17:** ERW: Support for interventions by age, gender, and Global South vs North

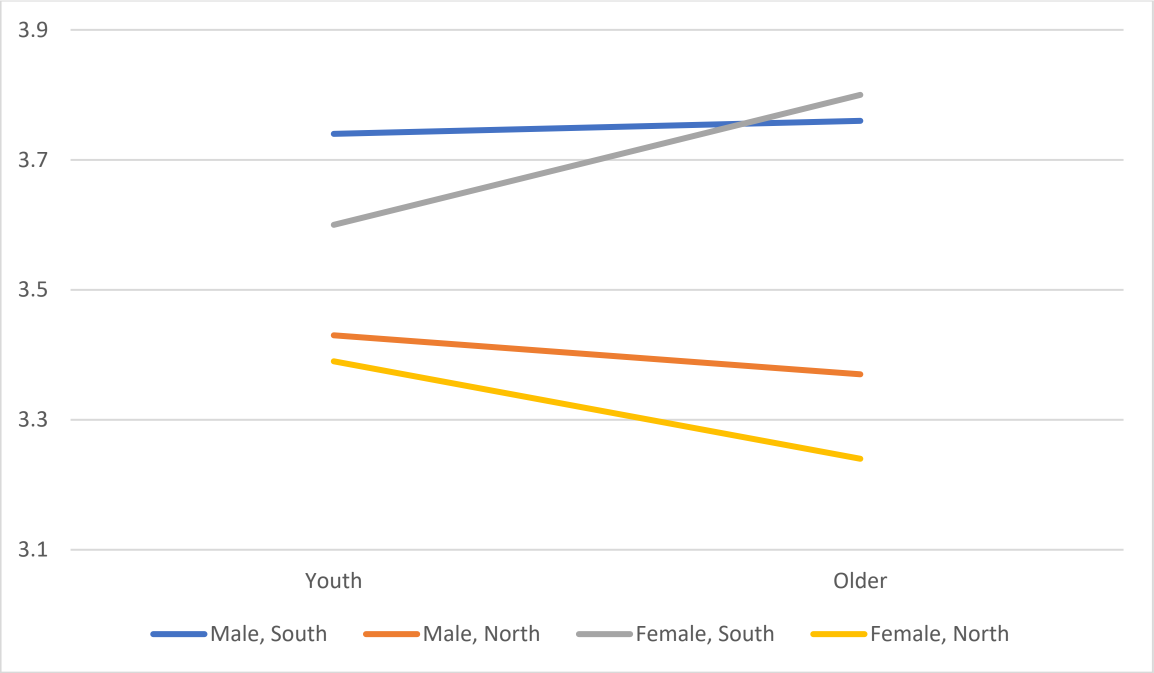


* In a three-way ANOVA, Global South, gender, the interaction between Global South by youth, and the three-way interaction were significant, but youth and the two remaining two-way interaction effects were non-significant.

**Supplementary Figure 18**: Biochar: Support for interventions by age, gender, and Global South vs North

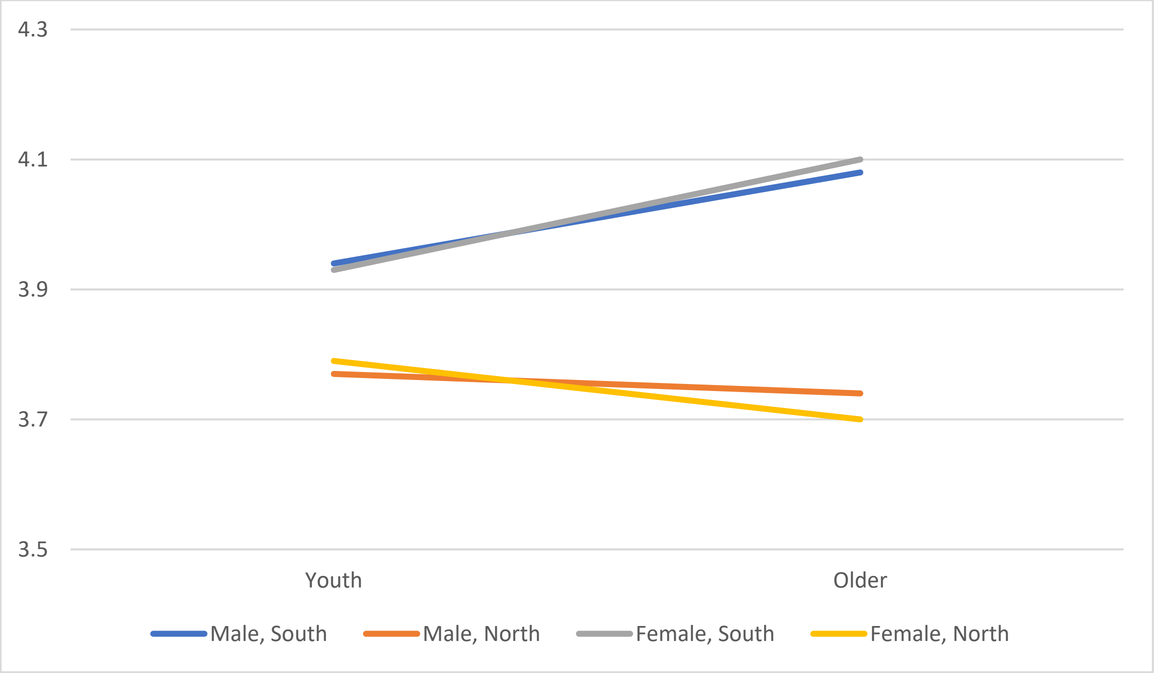


* In a three-way ANOVA, Global South and the interaction between Global South by youth were significant, but youth, gender, the two remaining two-way interaction effects, and the three-way interaction were non-significant.

Supplementary References


Braun, C., Merk, C., Pönitzsch, G., Rehdanz, K., & Schmidt, U. (2018). Public perception of climate engineering and carbon capture and storage in Germany: Survey evidence. *Climate Policy*, *18*(4), 471–484. https://doi.org/10.1080/14693062.2017.1304888

Cox, E., Spence, E., & Pidgeon, N. (2020). Public perceptions of carbon dioxide removal in the United States and the United Kingdom. *Nature Climate Change*, *10*(8), 744-749.

Da-Allada, C. Y., Baloïtcha, E., Alamou, E. A., Awo, F. M., Bonou, F., Pomalegni, Y., ... & Irvine, P. J. (2020). Changes in west African summer monsoon precipitation under stratospheric aerosol geoengineering. *Earth's Future*, *8*(7), e2020EF001595.

Dooley, K., Harrould-Kolieb, E., & Talberg, A. (2021). Carbon-dioxide Removal and Biodiversity: A Threat Identification Framework. *Global Policy*, *12*, 34-44.

Feldman, L., & Hart, P. S. (2021). Upping the ante? The effects of "emergency" and "crisis" framing in climate change news. *Climatic Change*, *169*(1-2), 10.

Fuss, S., Lamb, W. F., Callaghan, M. W., Hilaire, J., Creutzig, F., Amann, T., ... & Minx, J. C. (2018). Negative emissions—Part 2: Costs, potentials and side effects. *Environmental Research Letters*, *13*(6), 063002.

Gregory, R., & Lichtenstein, S. (1994). A hint of risk: tradeoffs between quantitative and qualitative risk factors. *Risk Analysis*, *14*(2), 199-206.

Jobin, M., & Siegrist, M. (2020). Support for the Deployment of Climate Engineering: A Comparison of Ten Different Technologies. *Risk Analysis*, *40*(5), 1058–1078. https://doi.org/10.1111/risa.13462

Krishnamohan, K. S., & Bala, G. (2022). Sensitivity of tropical monsoon precipitation to the latitude of stratospheric aerosol injections. *Climate Dynamics*, *59*(1-2), 151-168.

Liu, Z., Lang, X., Miao, J., & Jiang, D. Impact of Stratospheric Aerosol Injection on the East Asian Winter Monsoon. *Geophysical Research Letters*, e2022GL102109.

Low, S., Baum, C. M., & Sovacool, B. K. (2022). Taking it outside: exploring social opposition to 21 early-stage experiments in radical climate interventions. *Energy Research & Social Science*, *90*, 102594.

MacMartin, D. G., Ricke, K. L., & Keith, D. W. (2018). Solar geoengineering as part of an overall strategy for meeting the 1.5°C Paris target. *Philosophical Transactions of the Royal Society A: Mathematical, Physical and Engineering Sciences*, *376*(2119), 20160454. https://doi.org/10.1098/rsta.2016.0454

Pidgeon, N. F., & Spence, E. (2017). Perceptions of enhanced weathering as a biological negative emissions option. *Biology Letters*, *13*(4), 20170024. https://doi.org/10.1098/rsbl.2017.0024

Ricke, K., & Moreno-Cruz, J. (2022). 9.03 - Geo-Wedges: A Portfolio Approach to Geoengineering the Climate. In T. M. Letcher (Ed.), *Comprehensive Renewable Energy (Second Edition)* (pp. 14–24). Elsevier. https://doi.org/10.1016/B978-0-12-819727-1.00008-X

Rozin, P., & Royzman, E. B. (2001). Negativity bias, negativity dominance, and contagion. *Personality and Social Psychology Review*, *5*(4), 296-320.

Satterfield, T., Nawaz, S., & St-Laurent, G. P. (2023). Exploring public acceptability of direct air carbon capture with storage: Climate urgency, moral hazards and perceptions of the 'whole versus the parts.' *Climatic Change*, *176*(2), 14. https://doi.org/10.1007/s10584-023-03483-7

Sovacool, B. K. (2021). Reckless or righteous? Reviewing the sociotechnical benefits and risks of climate change geoengineering. *Energy Strategy Reviews*, *35*, 100656.

Steentjes, K., Pidgeon, N., Poortinga, W., Corner, A., Arnold, A., Böhm, G., Mays, C., Poumadère, M., Ruddat, M., Scheer, D., Sonnberger, M., Tvinnereim, E. (2017). European Perceptions of Climate Change: Topline findings of a survey conducted in four European countries in 2016. Cardiff, UK: Cardiff University

Sweet, S. K., Schuldt, J. P., Lehmann, J., Bossio, D. A., & Woolf, D. (2021). Perceptions of naturalness predict US public support for Soil Carbon Storage as a climate solution. *Climatic Change*, *166*(1), 22. https://doi.org/10.1007/s10584-021-03121-0

Tversky, A., and Kahneman, D. (1974). Judgment under Uncertainty: Heuristics and Biases. *Science*, *185*(4157), 1124–1131.

Van der Werff, E., Steg, L., & Keizer, K. (2013). The value of environmental self-identity: The relationship between biospheric values, environmental self-identity and environmental preferences, intentions and behaviour. *Journal of Environmental Psychology*, *34*, 55-63.

Wolske, K. S., Raimi, K. T., Campbell-Arvai, V., & Hart, P. S. (2019). Public support for carbon dioxide removal strategies: The role of tampering with nature perceptions. *Climatic Change*, *152*(3), 345–361. https://doi.org/10.1007/s10584-019-02375-z

Wright, M. J., Teagle, D. A. H., & Feetham, P. M. (2014). A quantitative evaluation of the public response to climate engineering. *Nature Climate Change*, *4*(2), 106-110. https://doi.org/10.1038/nclimate2087
