# Supplementary material for: Demographics shape public preferences for carbon dioxide removal and solar geoengineering interventions across 30 countries
Source: Commun Earth Environ. 2024 Oct 29;5(1):642. doi: 10.1038/s43247-024-01800-1 (PMC11522002; doi:10.1038/s43247-024-01800-1)
Supplement: Supplementary file 3 — Reporting Summary [file 43247_2024_1800_MOESM3_ESM.pdf]

nature portfolio

Corresponding author(s): Benjamin Sovacool, COMMSENV-23-1535C

Last updated by author(s): Aug 28, 2024

Reporting Summary

Nature Portfolio wishes to improve the reproducibility of the work that we publish. This form provides structure for consistency and transparency in reporting. For further information on Nature Portfolio policies, see our Editorial Policies and the Editorial Policy Checklist.

Statistics

For all statistical analyses, confirm that the following items are present in the figure legend, table legend, main text, or Methods section.

n/a	Confirmed	
☐	☒	The exact sample size (*n*) for each experimental group/condition, given as a discrete number and unit of measurement
☐	☒	A statement on whether measurements were taken from distinct samples or whether the same sample was measured repeatedly
☐	☒	The statistical test(s) used AND whether they are one- or two-sided<br>*Only common tests should be described solely by name; describe more complex techniques in the Methods section.*
☐	☒	A description of all covariates tested
☐	☒	A description of any assumptions or corrections, such as tests of normality and adjustment for multiple comparisons
☐	☒	A full description of the statistical parameters including central tendency (e.g. means) or other basic estimates (e.g. regression coefficient) AND variation (e.g. standard deviation) or associated estimates of uncertainty (e.g. confidence intervals)
☐	☒	For null hypothesis testing, the test statistic (e.g. *F*, *t*, *r*) with confidence intervals, effect sizes, degrees of freedom and *P* value noted<br>*Give P values as exact values whenever suitable.*
☒	☐	For Bayesian analysis, information on the choice of priors and Markov chain Monte Carlo settings
☒	☐	For hierarchical and complex designs, identification of the appropriate level for tests and full reporting of outcomes
☐	☒	Estimates of effect sizes (e.g. Cohen's *d*, Pearson's *r*), indicating how they were calculated

Our web collection on statistics for biologists contains articles on many of the points above.

Software and code

Policy information about availability of computer code

Data collection	We contracted with a professional online panel provider who used its own proprietary software to collect the survey data. We received data files from the survey company in SPSS format (version 29).
Data analysis	All analyses were done in IBM SPSS, version 29.

For manuscripts utilizing custom algorithms or software that are central to the research but not yet described in published literature, software must be made available to editors and reviewers. We strongly encourage code deposition in a community repository (e.g. GitHub). See the Nature Portfolio guidelines for submitting code & software for further information.

Data

Policy information about availability of data

All manuscripts must include a data availability statement. This statement should provide the following information, where applicable:

- Accession codes, unique identifiers, or web links for publicly available datasets
- A description of any restrictions on data availability
- For clinical datasets or third party data, please ensure that the statement adheres to our policy

The datasets used and analysed during the current study are available from the corresponding author upon reasonable request.

Research involving human participants, their data, or biological material

Policy information about studies with human participants or human data. See also policy information about sex, gender (identity/presentation), and sexual orientation and race, ethnicity and racism.

Reporting on sex and gender	As part of the analysis on public perceptions of carbon removal and solar radiation modification options, we consider the demographic dimension of gender (along with youth and age, poverty and income, as well as intersections and interactions between these categories). We exclusively use the term "gender" to reflect that this is shaped by social and cultural circumstances. Responses to this question item were based on self-reported data; importantly, participants were afforded the option of "Other/Prefer not to say". Given the absolutely small number of participants identifying in this way, such individuals were characterized as "missing data" for the purposes of the analysis.<br><br>Also, especially in view of the importance of issues of gender identity, we paid close attention to how the question about one's gender was framed in the languages. A handful of initial translations (e.g., Swedish, Norwegian') did not ask about gender but rather something to the effect of "Are you a man or a woman?". Translations were thus changed to a more neutral form, i.e., "What is your gender?". Similarly, in languages (like, German) where certain actors (e.g., climate activists or neighbors) can have both a masculine and feminine form, we made sure to include both of these in the answer option.<br><br>Sample sizes for analysis on support for each of the options are as follows (note: these may vary slightly given (i) differences in random assignment to categories with (ii) variation in those responding "Other/Prefer not to say" and (iii) heterogeneity due to individuals selecting "don't know" for the question on support - this was coded as a missing value for the resulting analyses): SAI (female = 4981, male = 4924), MCB (female = 4990, male = 4925), Space (female = 4984, 4923), Afforest (female = 4954, male = 5012), Soil Carbon (female = 4937, male = 4999), Blue Carbon (female = 4931, male = 4986), DACCS (female = 4918, male = 4967), BECCS (female = 4920, male = 4971), ERW (female = 4917, male = 4966), Biochar (female = 4920, male = 4967).<br><br>Regarding consent, full and informed consent was given by all participants before the beginning of the study, along with all participants being notified about the fact that their data would be handled in a fully anonymous manner and in complete accordance with the General Data Protection Regulation and any other pertinent data-security regulations, that any data would be analyzed in an aggregate fashion and would not be personally identifiable in any way, and that they had the right to withdraw their participation at any time.
Reporting on race, ethnicity, or other socially relevant groupings	The current manuscript did not employ socially relevant groupings for analysis, such as race or ethnicity. Besides gender, which we discussed above, we also examined the demographic dimensions of youth/age and poverty/income. Both of these relied on quantitatively set thresholds, while we also discussed and explained our specific "positionality" within the manuscript itself.
Population characteristics	Analysis in this paper considered a few different demographic dimensions: gender (self-reported); age and youth; poverty and income. Given that this information was gathered using survey items, the nature of the current research is not invasive; in any case, as noted in the supplemental materials, any questions about particular data being sensitive, including those that emerged in the course of the survey(s), were handled by erring on the side of caution and not asking a question in a given market. For instance, from the outset we decided not to ask about "political views" in China and the question on whether one self-identified as a "member of an ethnic minority or indigenous group" was removed in Estonia following feedback from participants. Upon reflection, this question seemed to be problematic for some survey participants given the Russo-Ukraine War and the presence of a sizable Russian ethnic minority group in Estonia. This question was thus removed for reasons of politically sensitivity and not wanting to irritate survey participants.<br><br>We also ensured that participants were generally afforded an "opt-out" option should they prefer not to answer.
Recruitment	Data collection was administered online by Norstat on behalf of Aarhus University using quota sampling, with informed consent obtained from all participants included in the study, all of whom were notified in advance of how their data would be handled as well as their right to withdraw at any time, and with all data delivered to researchers de-identified and anonymized by Norstat. Surveys were nationally representative of country populations 18-74 in terms of age, gender, and geographic region along with broad quotas for education and income (above versus below median for each country). Upon successful completion of the survey, participants received monetary compensation directly from the professional survey firm, Norstat.
Ethics oversight	All components of the research were granted ethical approval by relevant authorities at Aarhus University (#2021-13)

Note that full information on the approval of the study protocol must also be provided in the manuscript.

Field-specific reporting

Please select the one below that is the best fit for your research. If you are not sure, read the appropriate sections before making your selection.

☐ Life sciences ☒ Behavioural & social sciences ☐ Ecological, evolutionary & environmental sciences

For a reference copy of the document with all sections, see nature.com/documents/nr-reporting-summary-flat.pdf

Behavioural & social sciences study design

All studies must disclose on these points even when the disclosure is negative.

Study description	Quantitative study which uses nationally representative data from a large-scale, cross-country survey exercise taking place in 30 countries and 19 languages around the world, including in both the Global North and Global South, to assess public perceptions of climate intervention technologies (i.e., solar radiation modification and carbon dioxide removal) focusing on the explanatory relevance of demographic dimensions (gender, youth and age, poverty and income as well as interactions and intersections between them).
Research sample	The total sample consisted of 30,284 participants with at least 1,000 in each of the 30 countries surveyed. Surveys were nationally representative of country populations 18-74 in terms of age gender, and geographic region along with broad quotas for education and income. This approach was undertaken to ensure that the survey results, as much as possible, reflected perceptions of the countries in question, e.g., avoiding a bias towards the wealthiest or most highly educated. The only departures from national representativeness were: in Singapore, for those 55-74; in Greece, Switzerland, India, Dominican Republic, Chile, India, Saudi Arabia, Indonesia, for those 65-74; in Saudi Arabia, Indonesia, Chile, for some living in smaller regions; and in the United Kingdom, for those in North East England. Demographic characteristics for countries are available on request from authors.
Sampling strategy	Data collection was administered online by Norstat on behalf of Aarhus University using quota sampling to ensure national representativeness of country populations 18-74 in terms of age gender, and geographic region along with broad quotas for education and income. Given the novelty of the topic and the fact that many of the countries had never been surveyed in this context before, we aimed to have as large of a sample as possible while also achieving a broad scope in terms of countries included. In addition, given that participants would be assigned to one of three groups, more than 300 participants would be needed to have a power of 0.70-0.80 for a Cohen's d effect size which is small in nature (i.e., 0.2).
Data collection	Data collection employed an online survey design administed by Norstat and consisting of randomized assignment to one of the three technology types (solar radiation management, nature-based carbon removal, engineered carbon removal) and measures related to risks and benefits, support for different types of activities, support for various policy options along with questions on sociodemographic characteristics, beliefs about climate change and environment, and trust in institutions and actors and credibility of sources of information. Survey design, especially the information texts that were provided to participants, was revised and finalized by extensive piloting, input from external researchers, professional translators, and programmers and survey experts at Norstat, and drawing on the of results a soft launch undertaken for each country. Upon successful completion of the survey, participants received monetary compensation directly from the professional survey firm, Norstat.
Timing	Data collection for the surveys proceeded in stages for the thirty countries from August to December 2022.
Data exclusions	No data was excluded from the current study - prospective participants were however removed (and replaced) before the final data was delivered (by Norstat) in line with a set of pre-established quality checks throughout the survey.<br><br>. First, there were two comprehension checks though, based on results from the soft launch and given unfamiliarity of the topic, we opted not to exclude participants that failed to answer the question correctly in two attempts. Conversely, if someone answered the second (true/false) question wrong twice, then were removed, taking this as a good indicator of lack of attention. Second, there were two trap questions included in the survey, e.g., where participants were instructed to select “Strongly agree” – if individuals answered both questions incorrectly, they were also removed. Ultimately, we opted against removing participants if they only answered one trap question wrong given that the survey entailed lots of novel information, which also could be quite complex. Third, a “speeder flag” was instituted whereby participants who completed the survey in less than one-third of the median length of survey for a particular country were removed – in addition, for countries where the median length of survey was quite low, i.e., less than seven minutes, such individuals were also often removed and replaced, given questions of how realistic such a completion time would be given the minimum time constraints imposed for reading the information texts. Fourth, an open-ended question was included at the end of the survey, both to inquire about any possible problems and thereby make improvements to the survey and, in the case of problematic answers, as a reason to exclude participants. Lastly, at an administrative level, those with duplicate IP addresses and/or geolocation data which was, for whatever reason, not valid for a specific country.
Non-participation	Participants were told, at the stage of providing full and informed consent before the beginning of the study, that they had the right to withdraw their participation at any time, or indeed not to proceed with the survey. As only completed responses were provided to researchers by the professional survey firm in a de-identified and anonymized form, we are not aware of those who might have chosen not to participate, for whatever reason.
Randomization	Participants were randomly assigned to provide responses related to one of the three technology types: solar radiation management, ecosystem-based carbon dioxide removal, and engineered carbon dioxide removal. This assignment was done in a purely randomized manner by the professional survey firm, Norstat.

Reporting for specific materials, systems and methods

We require information from authors about some types of materials, experimental systems and methods used in many studies. Here, indicate whether each material, system or method listed is relevant to your study. If you are not sure if a list item applies to your research, read the appropriate section before selecting a response.

Materials & experimental systems

n/a	Involved in the study
☒	☐ Antibodies
☒	☐ Eukaryotic cell lines
☒	☐ Palaeontology and archaeology
☒	☐ Animals and other organisms
☒	☐ Clinical data
☒	☐ Dual use research of concern
☒	☐ Plants

Methods

n/a	Involved in the study
☒	☐ ChIP-seq
☒	☐ Flow cytometry
☒	☐ MRI-based neuroimaging

Plants

Seed stocks	N/A
Novel plant genotypes	N/A
Authentication	N/A
